# Relationship between urinary neutrophil gelatinase-associated lipocalin and selected biochemical and urinary parameters in dogs naturally infected with *Leishmania infantum*

**DOI:** 10.14202/vetworld.2024.2967-2974

**Published:** 2024-12-26

**Authors:** Daniela Proverbio, Roberta Perego, Luciana Baggiani, Eva Spada

**Affiliations:** Department of Veterinary Medicine and Veterinary Science, University of Milan, via dell’Università 6, 26900 Lodi, Italy

**Keywords:** canine leishmaniasis, canine, hematological parameters, proteinuria, urinary neutrophil gelatinase-associated lipocalin, urinary protein-to-creatinine

## Abstract

**Background and Aim:**

*Leishmania infantum* infection in dogs has several clinical manifestations. Glomerulonephritis, caused by circulating immune complexes, may cause proteinuria and progress to kidney failure, which is the primary cause of death in dogs with canine leishmaniasis (CanL). Renal proteinuria can be monitored in dogs with CanL for the early detection of renal involvement. Neutrophil gelatinase-associated lipocalin (NGAL) is a neutrophil-derived protein that is filtered by glomeruli and reabsorbed by proximal tubular cells. Urinary NGAL (uNGAL) is a sensitive marker of acute and chronic kidney disease in dogs. This study aimed to evaluate uNGAL concentrations in dogs naturally affected by CanL, to determine whether uNGAL concentration differs depending on the stage of disease based on the LeishVet and International Renal Interest Society (IRIS) classification systems, to compare uNGAL concentration with selected urinary and biochemical parameters related to kidney function, and to assess the clinicopathological status of dogs affected by CanL.

**Materials and Methods:**

We assessed uNGAL concentrations in 37 privately owned dogs naturally affected by CanL, in which urinary tract infections were excluded based on negative urine culture. No dog exhibited clinical signs related to impaired renal function. uNGAL concentration evaluated in dogs affected by CanL was compared to the one previously analyzed in the control group. Furthermore, the uNGAL concentration was compared between leishmaniasis dogs with biochemical and urinary parameters inside or outside the normal range and between dogs with different clinical stages of leishmaniasis based on the LeishVet clinical staging guidelines and IRIS classification.

**Results:**

The median uNGAL concentration in affected dogs was 50.2 ng/mL, which was significantly higher than that in healthy dogs (9.74 ng/mL [p = 0.0025]). uNGAL concentration was significantly higher in proteinuric leishmaniosis dogs than in non-proteinuric leishmaniosis dogs (p = 0.0001). Dogs classified as LeishVet clinical stage III had a higher mean uNGAL concentration than those classified as stage II (p = 0.0001) and median uNGAL concentration was statistically higher in dogs classified as IRIS stage 1 than in dogs affected by CanL with no clinical and pathological signs of renal disease. The amount of proteinuria and urinary sediment hyaline cast per high-power field of the microscope and total serum protein concentrations were significantly correlated with uNGAL concentration.

**Conclusion:**

To the best of our knowledge, only a few studies have measured uNGAL in dogs naturally affected by CanL. Although limited by the small number of cases, this study highlighted a significant increase in uNGAL levels in affected dogs compared with healthy dogs and confirmed the correlation between proteinuria and urinary excretion of uNGAL in dogs with leishmaniasis. This suggests that uNGAL can be used as a marker of kidney damage in dogs affected by CanL.

## Introduction

Canine leishmaniasis (CanL) is a major type of zoonosis caused by the parasite *Leishmania infantum*, which is transmitted by phlebotomine sand flies [1]. *L. infantum* is globally distributed from southern Europe to central and South America, and dogs are considered the main reservoir of this parasite [2]. Infected dogs may exhibit a broad spectrum of clinical manifestations, ranging from self-limiting disease to severe illness [1]. Disease susceptibility is associated with a strong and inefficient humoral immune response and the formation of circulating immune complexes. The deposition of these complexes in tissues has been associated with vasculitis, uveitis, arthritis, dermatitis, and glomerulonephritis (GN) [1].

Immunocomplex-mediated GN may progress to renal failure, which is the principal cause of death in affected dogs, and proteinuria is the first clinicopathological finding of nephropathy associated with leishmaniasis [1]. Initially, infected dogs may develop glomerular proteinuria; however, as the glomerular disease progresses, functional or structural lesions in tubular cells appear, and tubular proteinuria may develop in conjunction with impaired renal function and serum creatinine and azotemia increase [3]. Complete urinalysis, including the urinary protein-to-creatinine ratio (UPC), combined with serum creatinine and serum symmetric dimethylarginine (sSDMA) concentrations, can be used to monitor renal damage in dogs with leishmaniasis and to classify affected dogs according to the International Renal Interest Society (IRIS) staging scheme [4].

Neutrophil gelatinase-associated lipocalin (NGAL) is a lipocain protein with a molecular weight of 25 kDa [5]. It is expressed by neutrophils, monocytes, and epithelial cells in the kidneys, liver, lungs, colon, and adipose tissue [6]. NGAL is an iron-carrier protein that mediates the innate immune response. Furthermore, NGAL has bacteriostatic action that prevents bacterial iron uptake [7] due to its ability to bind and inhibit bacterial siderophore.

Under physiological conditions, renal NGAL is filtered and reabsorbed by proximal tubular cells. Following kidney injury, there is decreased NGAL re-absorption and increased NGAL expression, resulting in increased NGAL concentrations in both serum and urine [8].

Human studies by Valette *et al*. [9] and Yilmaz *et al*. [10] suggest that during the regenerative processes after kidney injury, NGAL is induced in proximal tubular epithelial cells and is a highly predictive biomarker of acute kidney injury (AKI), chronic kidney diseases (CKDs), and urinary tract infections (UTI).

In dogs, urinary NGAL (uNGAL) is a sensitive marker of kidney injury in dogs affected by X-linked hereditary nephropathy, acute and CKD [11], and kidney damage after heatstroke [12, 13]. Furthermore, uNGAL levels appear to be an earlier biomarker than serum creatinine for detecting AKI in dogs [13].

Based on the diffuse renal involvement observed in CanL infections, recent studies by Navarro *et al*. [14] and De Lima Ruy Dias *et al*. [15] have evaluated the use of urinary markers to identify early renal malfunction. To the best of our knowledge, only two studies have evaluated the use of uNGAL as a biomarker of renal damage during CanL. de Lima Ruy Dias *et al*. [15] investigated two treatments for CanL and showed that the uNGAL concentration decreased significantly after treatment with a combination of miltefosine and allopurinol. A more recent study showed a positive correlation between uNGAL concentration and proteinuria and the presence of moderate glomerular lesions in non-azotemic dogs experimentally infected with *L. infantum* [5].

The aims of this study were as follows: (1) to evaluate uNGAL in CanL-treated dogs in different clinical stages of the disease (according to LeishVet and IRIS staging), (2) to assess whether uNGAL concentration differs depending on the different stages of disease based on the LeishVet and IRIS classification systems [4, 16], and (3) to compare uNGAL concentration with selected urinary and biochemical parameters related to kidney function and to assess the clinicopathological status of dogs affected by CanL [1].

## Materials and Methods

### Ethical approval and Informed consent

Based on the University of Milan’s animal use regulations, formal ethical approval was not required because samples were collected during routine follow-up checks. After obtaining informed consent from the dog owners, surplus urine samples were submitted for uNGAL analysis.

### Study period and location

The study was conducted from January- 2019 to April-2022 at the Veterinary Teaching Hospital of the Department of Veterinary Medicine, University of Milan, Italy.

### Dogs

This retrospective study was conducted on 37 privately owned dogs naturally affected by CanL and admitted to the Veterinary Teaching Hospital of the University of Milan.

In all dogs, the diagnosis of clinical leishmaniasis was made based on: clinical signs and clinicopathological changes on blood tests compatible with CanL, the serological presence of specific serum antibodies (Immunoglobulin G) detected by immunofluorescent antibody test (IFAT) (cutoff 1:80), and positive real-time reverse transcription polymerase chain reaction on lymph node or spleen aspirates [1]. Based on the available clinical and clinicopathological data, dogs were classified into clinical stages according to both the LeishVet clinical staging [16] and IRIS staging [4]. uNGAL concentrations were measured in all dogs, and UTI was excluded based on negative urine culture results in the same sample in which uNGAL was measured.

### Sample collection

Blood and urine samples were collected as part of the routine follow-up of dogs affected by CanL. Blood samples were collected from the cephalic vein into ethylene-diamine tetra acetic acid tubes (Nuova Aptaca Srl, Canelli, AT, Italy) and plain tubes (Vacuette; Preanalitica SRL. Caravaggio Italy) and serum was obtained through centrifugation for 10 min at 2500× *g*.

### Biochemical and urinary parameters evaluation

All routine complete blood counts and serum chemistry profiles, antibody tests by IFAT, urinalysis, urine sediment preparations, and quantitative bacteriologic culture of urine were performed in the Veterinary Transfusion Research Laboratory (REVLab) and in the analysis laboratory of the Veterinary Teaching Hospital of the University of Milan. Quantitative polymerase chain reaction was performed in a private laboratory. Serum and blood samples were refrigerated at 4°C and analyzed within 24 h of sampling. Creatinine (Jaffè kinetic method) and total protein (TP) (colorimetric biuret method; Hagen Diagnostica S.r.l., Via Pratese 13 Firenze) analytes were evaluated using a Cobas Mira Classics Roche automated chemistry analyzer (Roche S.p. A., Mannheim, Germany) with reagents provided by Roche Diagnostics. Protein fractions were analyzed using a semiautomated agarose gel electrophoresis system with Hydragel Kit β1-β2 (SEBIA, Issy-les-Moulineaux, France) according to the manufacturer’s instructions. Protein fractions were determined as the percentage of optical absorbance and the absolute concentration (g/dL) was calculated automatically from the total serum protein concentration. Albumin/globulin (A/G) ratios were also calculated.

Before analysis, the urine samples were divided into three aliquots. Routine urinalysis was performed in the first aliquot within 2 h of collection, and protein and creatinine concentrations were measured in the supernatant using a Cobas Mira Classic chemistry analyzer (Roche Diagnostic Division, Basel, Switzerland). Urine protein and creatinine concentrations were determined using a Pyrogallol red colorimetric assay (Ben srl Biochemical Enterprise, Milan, Italy) and the modified kinetic Jaffe method (Hagen Diagnostica srl, San Giovanni [AR] Italy). The protein/creatinine ratio (UPC) was calculated automatically by dividing the urine protein concentration (mg/dL) by the creatinine concentration (mg/dL). Microscopic examination (10 fields at 400×) of the stained sediment from centrifuged urine was used to identify and quantify the mean numbers of leukocytes, red blood cells, epithelial cells, hyaline casts (HCs), and granular casts (GC) in the high-power field (hpf). The second aliquot was used for urine culture within 4 h of sample collection. The remaining aliquot of each sample was centrifuged at 309× *g* for 5 min, and the supernatant was removed, frozen, and stored at −20°C until uNGAL was determined.

### uNGAL determination

uNGAL levels were determined using a commercially available sandwich enzyme-linked immunosorbent assay (ELISA) kit (Dog NGAL ELISA kit, Bioporto Diagnostics, Denmark), validated for dogs. Assays were used in accordance with the manufacturer’s instructions. Briefly, after thawing, urine sample aliquots were diluted, and either samples or standards (NGAL, 0–400 ng/mL) were added in duplicate to 96-well plates and incubated between 20°C and 22°C (room temperature) for 60 min. After incubation and washing, biotinylated Dog-NGAL antibodies were dispensed into microwells and incubated at room temperature for 60 min. After washing, horseradish peroxidase-conjugated streptavidin was added and incubated at room temperature for 60 min. Finally, after washing, a color-forming Tetramethyl-Benzidine chromogenic substrate (TMB) was added, and after 10 min of incubation, the reaction was stopped with sulfuric acid and immediately read. The absorbance was measured at 450 nm. The concentration of NGAL in each sample was calculated from a standard curve obtained from a 4-parameter regression logistic curve. The detection limit of the ELISA was between 4 and 400 ng/mL. For statistical analyses, values below the detection limit were set at 0.2 ng/mL. The normal values of uNGAL in healthy dogs were determined using the same methodology used in a previous study by Proverbio *et al*. [7], and these results were adopted as a reference range for healthy dogs.

### Statistical analysis

All parameters considered for the study were tested for normal distribution using the D’Agostino–Pearson normality test. Quantitative measurements were described using summary statistics mean ± standard deviation (SD) for normally distributed data or median and interquartile range (IQR), if data were not normally distributed. Spearman’s coefficient of rank correlation (rho) was used to evaluate the relationship between uNGAL concentration and the following urinary and biochemical parameters: serum TP and creatinine concentration, protein fractions alpha 1, alpha 2, beta 1, beta 2, and gamma globulins, expressed as a percentage on the electrophoretic pattern, A/G ratio, urinary specific gravity and pH value, proteinuria and creatinuria ratio (UPC), and the hpf mean number of HC and GC, epithelial cells, and leucocytes (white blood cells) in urinary sediment.

When five or more individuals had values of selected urinary and biochemical parameters outside the reference range, the statistical difference in uNGAL level between those with values outside the reference range and those with normal parameters was calculated using Mann–Whitney independent sample test. Mann–Whitney independent sample test was also used to compare mean uNGAL concentration between sexes, proteinuric and non-proteinuric dogs, dogs classified in LeishVet stage II and III, and between dogs classified in IRIS stage 1 and 2 and other dogs affected by CanL but with no signs of renal damage. Significance was set at p < 0.05. Statistical analyses were conducted using commercially available software (MedCalc^®^ Statistical Software version 20.112, MedCalc Software Ltd., City Ostend, Belgium).

## Results

The group of 37 dogs affected by CanL included 20 pure breed dogs (seven dachshunds, six Rhodesian ridgebacks, four basset hounds, two labrador retrievers, and one rottweiler) and 17 mongrels, eight males and 29 females, with ages ranging from 3 to 12 years (mean 6.6 95%, CI 5.8–7.4). No dog had clinical signs related to impaired renal function [3]. Thirteen of the 37 dogs were proteinuric (UPC >0.5). Using the LeishVet clinical staging [16], 23/37 and 14/37 dogs were classified as stage II (moderate disease) and stage III (severe disease), respectively, and according to the IRIS classification [4], 11/37 and 5/37 dogs were classified as stage 1 and 2, respectively.

The statistical results of the urinary and biochemical parameters of 37 dogs with leishmaniasis are presented in Tables-1 and 2, respectively.

**Table-1:** Results of urinary parameters of 37 dogs with canine leishmaniosis.

Urinary parameters	Median	Minimum–maximum	Interquartile range	Reference value
uNGAL* ng/mL	50.2	0.0–518.6	5.4–246.7	
UPC*	0.2	0.02–3.77	0.07–1.4	< 0.5
SG*	1044	1010–1060	1040–1055	1020–1060
pH*	6	5–9	6–7	5.5–7
Leucocytes*	2	0–100	0.5–3.2	< 5
Hyaline casts*	0	0–3	0–2.25	< 2
Granular casts*	0	0–3	0–2	< 2
Renal epithelial cells	2	0–3	0–2	< 2

UPC=Urinary protein-to-creatinine ratio, SG=Urinary specific gravity,

* Not normally distributed, uNGAL=Urinary neutrophil gelatinase-associated lipocalin

**Table-2:** Results of biochemical parameters of 37 dogs with canine leishmaniosis.

Biochemical parameters	Median* Mean°	Minimum-Maximum	SD°	Reference value
TP° (g/dL)	7.1	5.8–8.6	0.7	6–8
A/G°	0.8	0.5–1.1		1–1.35
Albumin° (%)	43.9	35.1–53.3	4.2	44–62
Gamma globulin° (%)	15.25	6.1–27.6	5.9	5.7–17
Alpha 1 globulin° (%)	2.8	1.2–4.3	0.7	2.3–4.2
Alpha 2 globulin* (%)	16.2	12.4–25.2	14.4–18.2	11.4–19
Beta 1 globulin° (%)	5.4	3.4–8.4	1.4	3.2–8.9
Beta 2 globulin° (%)	16.2	12.3–21.4	1.8	9.8–18.7
Creatinine° (mg/dL)	0.9	0.4–1.8	0.3	<1.5

TP=Total protein, A/G=Albumin and protein ratio, IRQ=Interquartile range (IQR 25–75), SD=Standard deviation,

* Not normally distributed, °Normally distributed

The median uNGAL concentration was 50.2 ng/mL (IQR 5.4–246.7 pg/mL), which was significantly higher than that found by the same authors in healthy dogs (median:9.74 ng/mL, interquartile range IQR:1.93–25.43 pg/mL, intra-assay and inter-assay variability coefficient of variation (CV) 3.4% and 10.39%, respectively) (p = 0.0025) [7]. No statistically significant difference was found in the median value of uNGAL between males and females (p = 0.77). The median uNGAL value was significantly higher in proteinuric dogs than non-proteinuric dogs (p = 0.0001). Dogs classified at LeishVet clinical stage III had a statistically higher (p = 0.0001) mean concentration of uNGAL (301.65, IQR 146.5–435.8) than dogs in stage II (10.7, IQR 3.63–51.47) and median uNGAL concentration was statistically higher (p = 0.0001) in dogs in stage 1 (306.65, IQR 169.07–473.6) of the IRIS classification than in dogs affected by CanL but without clinicopathological signs of kidney disease (10.7 IQR 3.35–38.6), whereas a statistically significant difference between uNGAL concentrations in stage IRIS I and II dogs was not observed.

**Figure-1:**
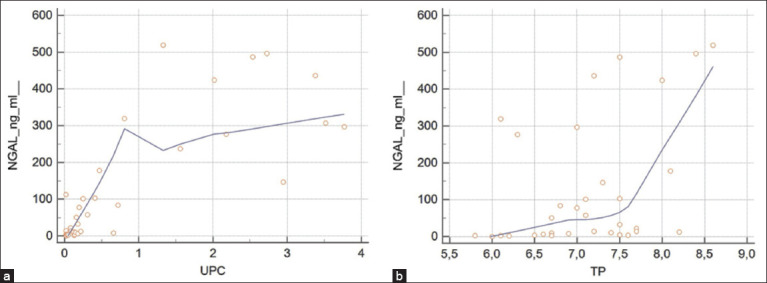
Graphic representation of Spearman correlation (a) between urinary neutrophil gelatinase-associated lipocalin (uNGAL) and urinary protein/creatinine and (b) between uNGAL and serum total protein concentrations.

Only the UPC value, HC number, and TP concentration were significantly correlated with uNGAL concentration: Table-3 and Figure-1.

**Table-3:** Correlation between selected urinary and biochemical variables and uNGAL concentration in 37 dogs with canine leishmaniosis.

Parameters	Spearman’s coefficient (rho)	Significance level*
uNGAL/UPC	0.814	**< 0.0001**
uNGAL/SG	0.027	0.8736
uNGAL/pH	0.190	0.2590
uNGAL/leucocytes	−0.088	0.6062
uNGAL/epithelial cells	0.201	0.2331
uNGAL/granular casts	0.081	0.6343
uNGAL/hyaline casts	0.487	**0.0023**
uNGAL/total protein	0.484	**0.0043**
uNGAL/albumin %	−0.143	0.4514
uNGAL/gamma-globulin ratio	−0.026	0.8878
uNGAL/alpha 1 globulin	−0.234	0.2050
uNGAL/2 globulin percentage	0.274	0.1357
uNGAL/beta 1%	0.214	0.2467
uNGAL/beta 2%	0.107	0.5670
uNGAL/A/G	−0.163	0.3811
uNGAL/creatinine	−0.231	0.1960

uNGAL=Urinary neutrophil gelatinase-associated lipocalin, UPC=Urinary protein/creatinine ratio, SG=Urinary specific gravity, TP=Total protein, A/G=Albumin, protein ratio.

* Not normally distributed, *p<0.05 is considered significant, bold values indicate statistically significance

The A/G ratio was below the normal reference range (28/32) in almost all dogs. Two of the 33 and 3/33 dogs had % alpha 2 and beta 2 fractions above the reference range, respectively, and in 4/33 dogs, the percentage of the alpha 1 fraction on the electrophoresis pattern was below the reference range. No dogs showed alterations in the beta 1 electrophoretic fraction.

**Figure-2:**
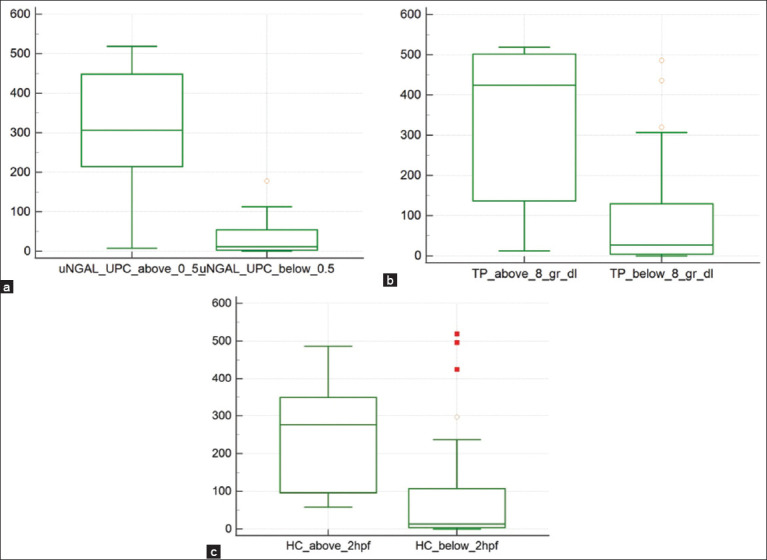
Box plot showing the difference between urinary neutrophil gelatinase-associated lipocalin concentration in dogs with (a) urinary protein/creatinine values above and below 0.5, (b) total protein concentration above and below 8 g/dl, and (c) hyaline cast high power field (hpf) mean number above and below 2 in urinary sediment. Data are presented as boxes and whiskers. Each box includes 25 and 75 interquartiles, whereas the line inside the box represents the median and the whiskers represent the minimum and maximum values.

Table-4 and Figure-2 show the statistical differences in uNGAL levels between dogs with values outside the reference range and those with normal parameters.

**Table-4:** Median, 95% confidence interval, minimum and maximum value, and p*-*value evaluated by Mann–Whitney independent sample test to compare mean uNGAL concentration in dogs with parameter values above, below, or within the normal range.

Parameter	Value	Median	95%CI	Min-Max	p-value	Number of dogs
TP g/dL	>8 g/dL	424.2	-	12.3–518.6	**0.0183**	5
	<8 g/dL	17.8	6.4–81.2	0–486.2		28
Albumin%	<44%	83.4	13.8–444.8	10.7–518.6	0.0793	11
	>44%	11.9	3.6–134.7	0–495.8		20
Gamma globulin%	>17%	32.2	12.54–389.8	10.7–518.6	0.2671	9
	<17%	32.1	3.7–147.9	0–495.8		22
UPC	>0.5	306.6	194.4–459.4	7.7–518.6	**< 0.0001**	13
	<0.5	11.5	3.7–36.8	0–178.1		25
Sediment leucocytes (hpf)	>5	54	2.5–210.7	1.8–486.2	0.6315	8
	<5	32.2	10.5–191.8	0–518.6		29
Sediment hyaline cast (hpf)	>2	276.3	80.7–419.7	57.9–486.2	**0.0037**	9
	<2	13.2	5–71.5	0–518.6		28
Sediment granular casts (hpf)	>2	32.2	2.7–302.2	1.8–424.2	0.5608	7
	<2	67.5	10.9–140.6	0–518.6		30
Sediment epithelial cells of the HPF	>2	32.1	3.12–401.4	2.5–424.2	0.9836	6
	<2	57.9	10.3–126.6	0–518.6		31

uNGAL=Urinary neutrophil gelatinase-associated lipocalin, UPC=Urinary protein/creatinine ratio, TP=Total protein, CI=Confidence interval, *p < 0.05 is considered significant, bold values indicate statistically significance

## Discussion

This study evaluated uNGAL levels in dogs naturally affected by *L. infantum*. In the study population, the median uNGAL concentration (50.2 ng/mL) was higher than that found in healthy dogs (9.74 ng/mL) by Proverbio *et al*. [7] while Hsu *et al*. [6], Steinbach *et al*. [18], and Daure *et al*. [17] found 0.24, 7.6, and 0.4 ng/mL, respectively. When considering dogs affected by CanL classified based on the LeishVet clinical staging and IRIS staging system, there was a statistically higher mean concentration of uNGAL in IRIS 1 dogs than in dogs affected by CanL but without clinicopathological signs of kidney disease and in dogs in LeishVet stage III than in dogs in LeishVet stage II. Notably, the mean uNGAL level in our population is similar to that reported by Steinbach *et al*. [18] in dogs with CKD (median 43.6 ng/mL). During kidney injury, there is a decrease in tubular NGAL reabsorption and an increase in its expression, resulting in an increased uNGAL concentration in the urine [6]. It is possible that the dogs in our study had kidney damage. In fact, 13/37 subjects were proteinuric, and 5/37 had serum creatinine levels between 1.8 and 2.4 mg/dL. Based on the results of kidney biopsy and diagnostic imaging, several authors have reported the presence of kidney lesions in dogs with CanL, even in the absence of proteinuria or azotemia [3, 19]. In dogs with CanL immunocomplex deposition on the glomerular membrane, two principal types of GN: focal or diffuse mesangial–proliferative GN and membranoproliferative GN [3], may progress to interstitial fibrosis, glomerulosclerosis, and renal failure [4, 18]. The detection of proteinuria is considered one of the first signs of renal damage in dogs [3, 17]. Therefore, in dogs affected by CanL assessment of proteinuria, the protein/creatinine ratio (UPC), with a cutoff value of UPC > 0.5, should be used to monitor renal function for the early detection of kidney dysfunction [3]. In our study, there was a strong positive correlation between uNGAL concentration and UPC value (rho = 0.814) and between uNGAL and urinary sediment HC numbers (rho = 0.417). Furthermore, the median uNGAL concentration was statistically significantly higher in dogs with UPC >0.5 and mean number of urinary sediment HCs >2/hpf than in dogs with normal values (p < 0.0001 and p = 0.0023, respectively). This finding is in accordance with a previous study in dogs treated for CanL where a correlation was found between mean UPC and uNGAL values before and after treatment [15]. The statistically significant differences found in the concentration of uNGAL between dogs affected by CanL but without clinicopathological signs of kidney disease and dogs in stage IRIS 1 and between dogs in LeishVet stage II and those in LeishVet stage III also correlated with the concentration of UPC and uNGAL since the classification of subjects is affected by proteinuria. Peris *et al*. [5] in a study of dogs experimentally infected with *L. infantum* found that uNGAL concentration was associated with proteinuria, glomerular lesions, and incidental tubulointerstitial tissue damage, suggesting that increasing uNGAL filtration during CanL infection may be associated with the early renal impairment, which mainly involves the glomerulus, even if failure of tubular resorption due to tissue or functional damage cannot be ruled out [5]. In our study, 7/37 dogs had GCs and 6/37 had epithelial cell numbers >2 hpf in urinary sediment. The presence of granular and cellular casts may be consistent with tubular lesions [20], suggesting the presence of tubular damage. In this study, 23/37 dogs were classified as stage II (moderate disease) according to the LeishVet clinical staging system and 14/37 were classified as stage III (severe disease), but no significant correlation was found between uNGAL and serum creatinine concentration. uNGAL indicates active lesions during renal damage. Although active damage increases in parallel with a decrease in renal function, the levels of uNGAL and serum creatinine may represent different functionalities [21]. In fact, unlike uNGAL, the concentration of serum creatinine is not a real-time indicator of renal lesions because it takes many days to reach a steady state between the production of serum creatinine and the decrease in the excretion of serum creatinine [22]. In addition, serum creatinine concentration increases only when 75% of kidney function has been lost [3]. Our study found a positive correlation between uNGAL concentration and TP serum concentration (p = 0.0023). Furthermore, the median uNGAL concentration was statistically significantly higher (p = 0.0183) in dogs with TP values above 8 g/dL than those with TP values below 8 g/dl. Dysproteinemia is the most common clinicopathological finding in dogs affected by CanL. It is generally characterized by hyperproteinemia secondary to hyperglobulinemia due to an increase in the gamma and beta and, less frequently, alpha-2 globulins with a consequent decrease in the A/G ratio [3]. In the 9/31 dogs evaluated in this study, an increase in TP was associated with an increase in gamma globulins. The excessive production of globulins results in the formation of immunocomplexes and, therefore, renal damage, which could explain the correlation between the uNGAL and TP serum concentrations found in our study, although no statistically significant correlation between gamma globulins and uNGAL was found. Almost all subjects showed a reduction in A/G and a weak but not significant correlation between A/G and uNGAL concentration. The decrease in the A/G ratio is a frequent finding during CanL, so much so that some authors suggest that it is a sensitive test for CanL [1], and hypoalbuminemia is considered a negative prognostic indicator in dogs with leishmaniasis [23]. In our study, 12/31 dogs had albumin levels below 44% on the electrophoretic pattern. Albumin is a negative acute phase protein (APP), and its serum concentration may be influenced by active inflammation, even if a quota could be lost due to proteinuria [3]. A decrease in albumin concentration is frequently observed during CanL. Although not statistically significant, the percentage of albumin showed a weak negative correlation with uNGAL, confirming that hypoalbuminemia can be related to or intensified by proteinuria [3]. The results of this study confirmed the correlation between proteinuria and urinary excretion of uNGAL in CanL -affected dogs. Because uNGAL is considered one of the main biomarkers of AKI in dogs [24], with increases detected earlier than serum creatinine, it could be used as an early marker of kidney damage in dogs affected by CanL. The clinical evaluation of proteinuria requires not only the determination of its magnitude but also localization, that is, whether glomerular or tubular. Numerous studies have evaluated alternative markers to avoid the need for renal biopsy, which is invasive and not feasible in many patients, to detect glomerular or tubular damage early in CanL [14]. If future studies confirm the promising results obtained in the evaluation of uNGAL in CanL, this parameter will contribute to the identification of a reliable urinary marker that provides information about the severity of renal damage earlier than traditional parameters such as serum creatinine concentration and proteinuria [25]. This study has some limitations: the first is the lack of follow-up of dogs over time and the failure to evaluate whether the change in uNGAL concentration occurred before proteinuria was detected. In addition, due to the retrospective nature of the study, some markers of renal damage, such as sSDMA, may be missed. Finally, APP, such as C-reactive protein and haptoglobin, which could support the monitoring of clinical cases of leishmaniasis, were not evaluated. Future research should evaluate the correlation between uNGAL and APPs, although the increase in APPs is not a specific marker of CanL [26]. Mild changes may be observed in subclinical forms, whereas high values can be observed during the acute phase of CanL and other pathologies. Further studies with a larger number of dogs monitored over time are needed to determine whether changes in uNGAL concentration precede increases in proteinuria as markers of renal injuries in dogs affected by CanL.

## Conclusion

To the best of our knowledge, only a few studies have measured uNGAL concentrations in dogs naturally affected by CanL. This study demonstrated a significant increase in uNGAL levels in dogs with renal damage compared with healthy controls, providing valuable evidence of uNGAL as a potential marker of kidney damage in dogs with CanL. The strength of this study lies in its correlation of uNGAL levels with proteinuria, urinary sediment hyaline casts, and total serum protein concentrations, as well as the assessment of clinical stages of CanL using both the LeishVet and IRIS classification systems. Despite being limited by the small sample size, these findings highlight the potential clinical utility of uNGAL for the early detection and monitoring of kidney damage in CanL, particularly in dogs at higher stages of the disease.

## Authors’ Contributions

DP: Conceptualization, data analysis, and validation. ES: Conceptualization and experimental design of the animal model, statistical analyses, and reviewed the manuscript. RP: Synthesis and interpretation of results. LB: Data collection, methodology, and experimental design for biochemical and urinary analysis. All authors have read and approved the final manuscript.
